# An approach to low-density polyethylene biodegradation by *Bacillus amyloliquefaciens*

**DOI:** 10.1007/s13205-014-0205-1

**Published:** 2014-03-17

**Authors:** Merina Paul Das, Santosh Kumar

**Affiliations:** Department of Industrial Biotechnology, Bharath University, Chennai, 600073 Tamil Nadu India

**Keywords:** Biodegradation, LDPE, *Bacillus amyloliquefaciens*, Degradation analysis

## Abstract

Low-density polyethylene (LDPE) is a major cause of persistent and long-term environmental pollution. In this paper, two bacterial isolates *Bacillus amyloliquefaciens* (BSM-1) and *Bacillus amyloliquefaciens* (BSM-2) were isolated from municipal solid soil and used for polymer degradation studies. The microbial degradation LDPE was analyzed by dry weight reduction of LDPE film, change in pH of culture media, CO_2_ estimation, scanning electron microscopy (SEM), and fourier transform infrared FTIR spectroscopy of the film surface. SEM analysis revealed that both the strains were exhibiting adherence and growth with LDPE which used as a sole carbon source while FTIR images showed various surface chemical changes after 60 days of incubation. Bacterial isolates showed the depolymerization of biodegraded products in the extracellular media indicating the biodegradation process. BSM-2 exhibited better degradation than BSM-1 which proves the potentiality of these strains to degrade LDPE films in a short span of time.

## Introduction

Plastic is a man-made hazardous long-chain synthetic polymer. The annual global demand for plastics has consistently increased over the recent years and presently stands at about 245 million tones. Being a versatile, lightweight, strong and potentially transparent material, plastics are ideally suited for a variety of applications. Their low cost, excellent oxygen/moisture barrier properties, bio-inertness and light weight make them excellent packaging materials (Andrady 2011). However, large amount of accumulation of plastic waste is leading a harmful effect on nature, causing environmental pollution as it is resistant to biodegradation. There are several chemicals within plastic material itself that have been added to give it certain properties such as Bisphenol A, phthalates and flame retardants. These all have known negative effects on human and animal health, mainly affecting the endocrine system. There are also toxic monomers, which have been linked to cancer and reproductive problems. Awareness of the waste problem and its impact on the environment has awakened new interest in the area of degradable polymers. The interest in environmental issues is growing and there are increasing demands to develop material which do not burden the environment significantly (Shah et al. 2008).

Low-density polyethylene (LDPE) is a widely used non-biodegradable thermoplastic. To deal with this environmental problem related to non-biodegradable thermoplastics, research to modify non-biodegradable thermoplastics to biodegradable materials is of great interest (Zheng et al. 2005). Furthermore, these synthetic polymers are normally not biodegradable until they are degraded into low molecular mass fragments that can be assimilated by microorganisms (Francis et al. 2010).

The biodegradable polymers are designed to degrade it quickly by microbes since microorganisms are capable of degrading most of the organic and inorganic materials, including lignin, starch, cellulose and hemicelluloses (Sadocco et al. 1997). In addition, biodegradable plastics offer a lot of advantages such as increased soil fertility, low accumulation of bulky plastic materials in the environment (which invariably will minimize injuries to wild animals), and reduction in the cost of waste management (Tokiwa et al. 2009). The microbial species associated with the degrading polymers were identified as bacteria (*Pseudomonas, Streptococcus, Staphylococcus, Micrococcus, Moraxella*), fungi (*Aspergillus niger, Aspergillus glaucus*), *Actinomycetes* sp. and *Saccharomonospora* genus (Swift 1997). Biodegradation has been considered as a natural process in the microbial world where polymers can be used as carbon and energy sources for their growth and plays a key role in the recycling of these materials in the natural ecosystem (Albertsson et al. 1987). The microbial degradation of plastics is caused by certain enzymatic activities that lead to a chain cleavage of the polymer into oligomers and monomers. These water soluble enzymatically cleaved products are further absorbed by the microbial cells where they are metabolized. Aerobic metabolism results in carbon dioxide and water (Starnecker and Menner 1996), whereas anaerobic metabolism results in carbon dioxide, water and methane as the end products, respectively (Gu et al. 2000). The aim of this research was to study the biodegradation of low-density polyethylene using various techniques in vitro by selected and potent microorganism isolated from municipal solid waste.

## Materials and methods

### Pre-treatment and preparation of LDPE powder

Low-density polyethylene (LDPE) was obtained from B.N. Polymers, Bangalore, India. LDPE films were cut into small pieces, immersed into xylene and boiled for 15 min, followed by crushing with blender at 3,000 rpm. As obtained LDPE powder was further washed with ethanol, dried overnight in hot air oven at 60 °C and stored at room temperature for further use.

### Polyethylene degrading bacteria and culture conditions

The bacteria used in this study, *B. amyloliquefaciens* (BSM-1) (GenBank accession no. KC924446) and *B. amyloliquefaciens* (BSM-2) (GenBank accession no. KC924447) (Das and Kumar 2013), were isolated from the municipal solid waste landfill area, Pallikaranai (12.9377N/80.2153E, 7 m above sea level), Chennai, India and maintained on nutrient agar at 4 °C. The polymer degrading bacteria were identified using synthetic media supplemented with 0.3 % LDPE powder. The synthetic media composition was as follows: (g/L: K_2_HPO_4_, 1; KH_2_PO_4_, 0.2; (NH_4_)_2_SO_4_, 1; MgSO_4_·7H_2_O, 0.5; NaCl, 1; FeSO_4_·7H_2_O, 0.01; CaCl_2_·2H_2_O, 0.002; MnSO_4_·H_2_O, 0.001; CuSO_4_·5H_2_O, 0.001; ZnSO_4_·7H_2_O, 0.001; Agar 15; pH 7.0).

### Biodegradation studies

Biodegradation tests were performed with 3 g samples of LDPE films (1.5 × 1.5 cm) that had been dried overnight at 60 °C, weighed, disinfested (30 min in 70 % ethanol), air-dried for 15 min in Laminar air flow chamber and added to Erlenmeyer flasks containing 300 ml of synthetic medium. LDPE degradation study was carried out using both the bacterial strains individually. Each flask inoculated with 3 ml of 24 h old culture (BSM-1 and BSM-2) grown on LDPE-supplemented medium was used as inoculums to avoid any associated long lag phase. Then cultures were incubated on a rotary shaker (Neolab Instruments) at 33.3 °C and 130 rpm for 60 days. Each test consisted of three replicates.

### Measurement of biodegradation

#### Determination of pH change

Study of pH change was adopted to make sure about any metabolic activity of the microbial strain in supplemented medium, as metabolism shown by microbial cells may greatly support the evidence of degradation. The pH of the each bacterial suspension was measured at an interval of 10 days during the study. The pH probe was inserted in the broth to measure the pH. Initial value of the medium was ensured to be 7 ± 0.3 for both strains using phosphate buffer.

#### Determination of dry weight of residual polymer

To facilitate accurate measurement of the weight of the residual polyethylene, the polyethylene sheets were recovered after the 60 days of incubation and washed off the bacterial biofilm from the polymer surface with a 2 % (v/v) aqueous sodium dodecyl sulfate solution for 4 h (using shaker), followed by distilled water and finally with 70 % ethanol to ensure maximum possible removal of cells and debris. The washed polymer pieces were placed on a filter paper and dried overnight at room temperature before weighing.

#### CO_2_ evolution test

The self-modified simple apparatus was designed which consists of control and test vessels and sterile air was supplied to the system for aeration. Here, the polymer incubated with microbes served as the test and polymer without microbes served as control. After incubation, both the metabolic and atmospheric CO_2_ from the test vessel and atmospheric CO_2_ from the control vessel were trapped and assessed using “Sturm test” (Sturm 1973) for each isolate.

#### Scanning electron microscopy (SEM)

The untreated and treated samples after 60 days of duration were subjected to SEM analysis (after washing with 2 % (v/v) aqueous SDS and distilled water repeatedly through mild shaking for few minutes and additionally flushed with 70 % ethanol with the objective of removal of cells so as to get maximum surface to be exposed for visualization. The samples were pasted onto the SEM Sample Stub using a carbon tape and sample was coated with gold for 40 s and analyzed under high-resolution scanning electron microscope (JEOL, Model JSM-6390LV).

#### FTIR analysis

Fourier transform infrared spectroscopic studies were carried out for control and bacteria-treated LDPE films. The analysis was performed using Perkin-Elmer Spectrum-One FTIR spectroscopy in the horizontal mode with thallium bromide disks.

## Results and discussion

Biodegradable plastic is a favorable solution of plastic disposal or accumulation problem in nature. As domestic and industrial waste containing huge amount of low-density polyethylene, in this work municipal landfill solid waste sample was collected to isolate the microorganisms which showed potent biodegradation. The bacterial isolates can grow in a LDPE-supplemented synthetic medium utilizing LDPE as sole carbon and energy sources. These observations indicate the formation and attachment of a biofilm on LDPE film. The microbial colonization on polymer surface is the first requirement for its biodegradation (Yabannavar and Bartha 1993).

### Biodegradation studies

After incubation period of 60 days, the degrading capability of the strains *Bacillus amyloliquefaciens* (BSM-1) and *Bacillus amyloliquefaciens* (BSM-2) was analyzed and interpreted using various parameters.

#### pH Change

**Fig. 1:**
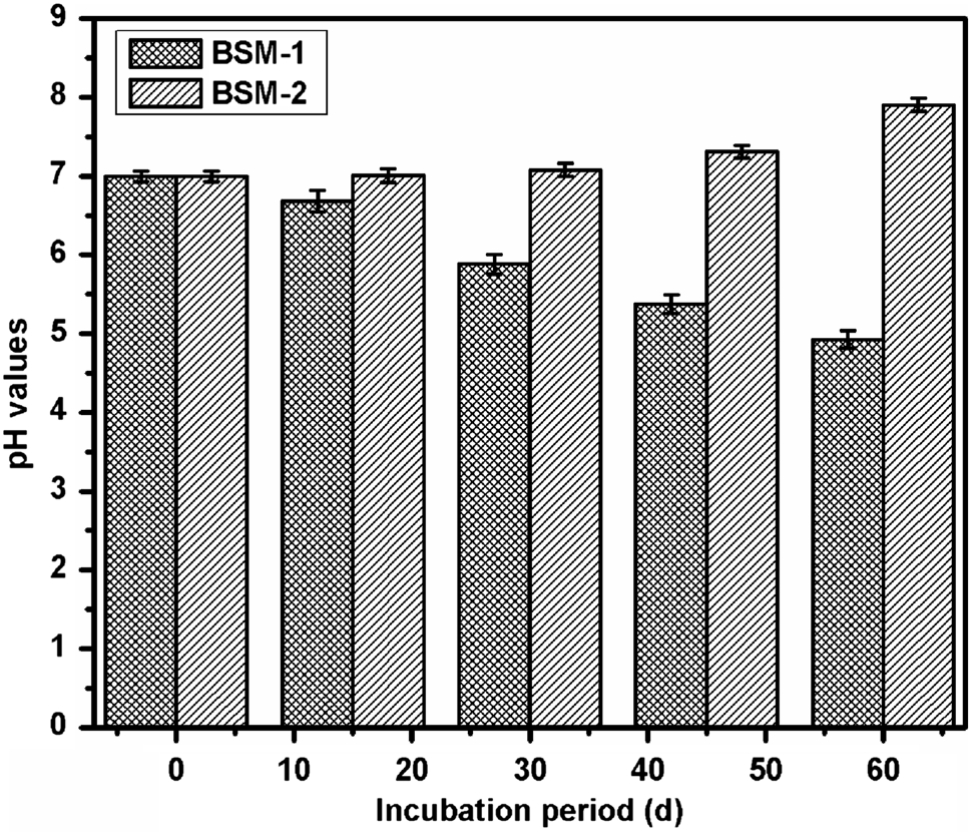
Variation in pH level during biodegradation due to microbial activity

Figure 1 shows the variation in pH of both bacterial suspensions during and after the biodegradation. Microorganisms secrete a variety of enzymes into the soil water, which begin the breakdown of the polymers. Two types of enzymes are involved in the process, namely intracellular and extracellular depolymerases. Exoenzymes from the microorganisms first breakdown the complex polymers giving short chains or monomers that are small enough to permeate through the cell walls to be utilized as carbon and energy sources by a process of depolymerization (Dey et al. 2012). Bacterial isolates, BSM-1 and BSM-2 showed the production of some enzymes and metabolites with the indication of pH change supporting the metabolic activity of strains on the LDPE substrate and also its degradation.

#### Weight reduction

**Fig. 2:**
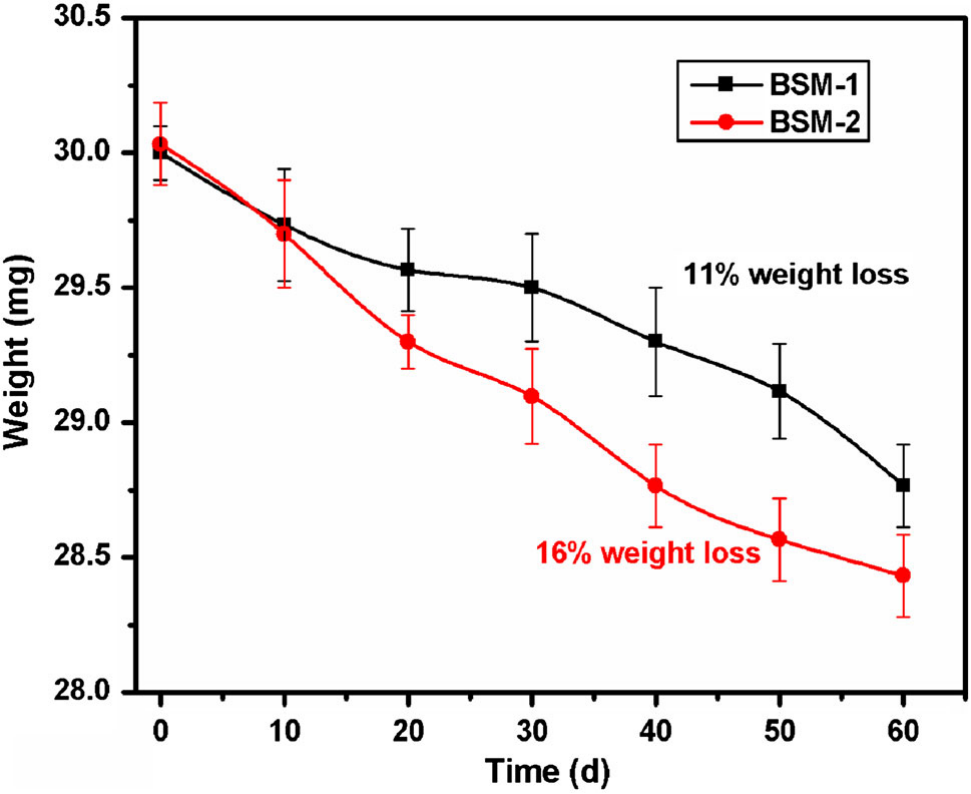
Degradation of LDPE films (initial weight: 30 mg with 1.5 × 1.5 cm) on synthetic medium inoculated with strain BSM-1 and BSM-2 incubated at 33.3 °C for 60 days

A simple and quick way to measure the biodegradation of polymers is by determining the weight loss. Microorganisms that grow within the polymer lead to an increase in weight due to accumulation, whereas a loss of polymer integrity leads to weight loss. Weight loss is proportional to the surface area since biodegradation usually is initiated at the surface of the polymer. After the degradation period, the LDPE films were treated with SDS as surfactant which denatured the cells and completely washed off from the surface. The reduction in weight was observed after the biodegradation of LDPE (Fig. 2).

#### Assessment of mineralization level

Sturm test is the method where the degradation was attributed to the amount of metabolic carbon dioxide evolved during the growth period. The polymers are made up of carbon chain and when it degrades through the microbes CO_2_ and H_2_O are obtained as byproducts, the process is called mineralization in which polymer is first converted to monomers by breaking the links and then to more simpler compounds to be assimilated into the living cells. The level of CO_2_ was calculated from the control (atmospheric CO_2_) and reaction chamber (atmospheric and metabolic CO_2_) after 60 days of biodegradation study. Theoretical carbon dioxide evolution for 3 % LDPE was estimated to be 11 g/L for complete biodegradation. Here, the percentage of biomineralization level of LDPE through evolved carbon dioxide from reaction chambers was calculated for strain BSM-1 and BSM-2 by comparing with the corresponding values of control chambers (Table 1). The result shows the potentiality of *Bacillus amyloliquefaciens* and supports the fact of biodegradation and biomineralization of this hazardous polymer.

**Table 1:** Biomineralization level of microbial isolates

Isolates	Carbon dioxide evolution (g/L)			Mineralization level (%)
	Control (atmospheric) (C)	Test (metabolic + atmospheric) (T)	T − C (metabolic)	
BSM-1	16.26	17.58	1.32	12
BSM-2	18.31	19.92	1.61	14.7

#### SEM analysis of LDPE film

**Fig. 3:**
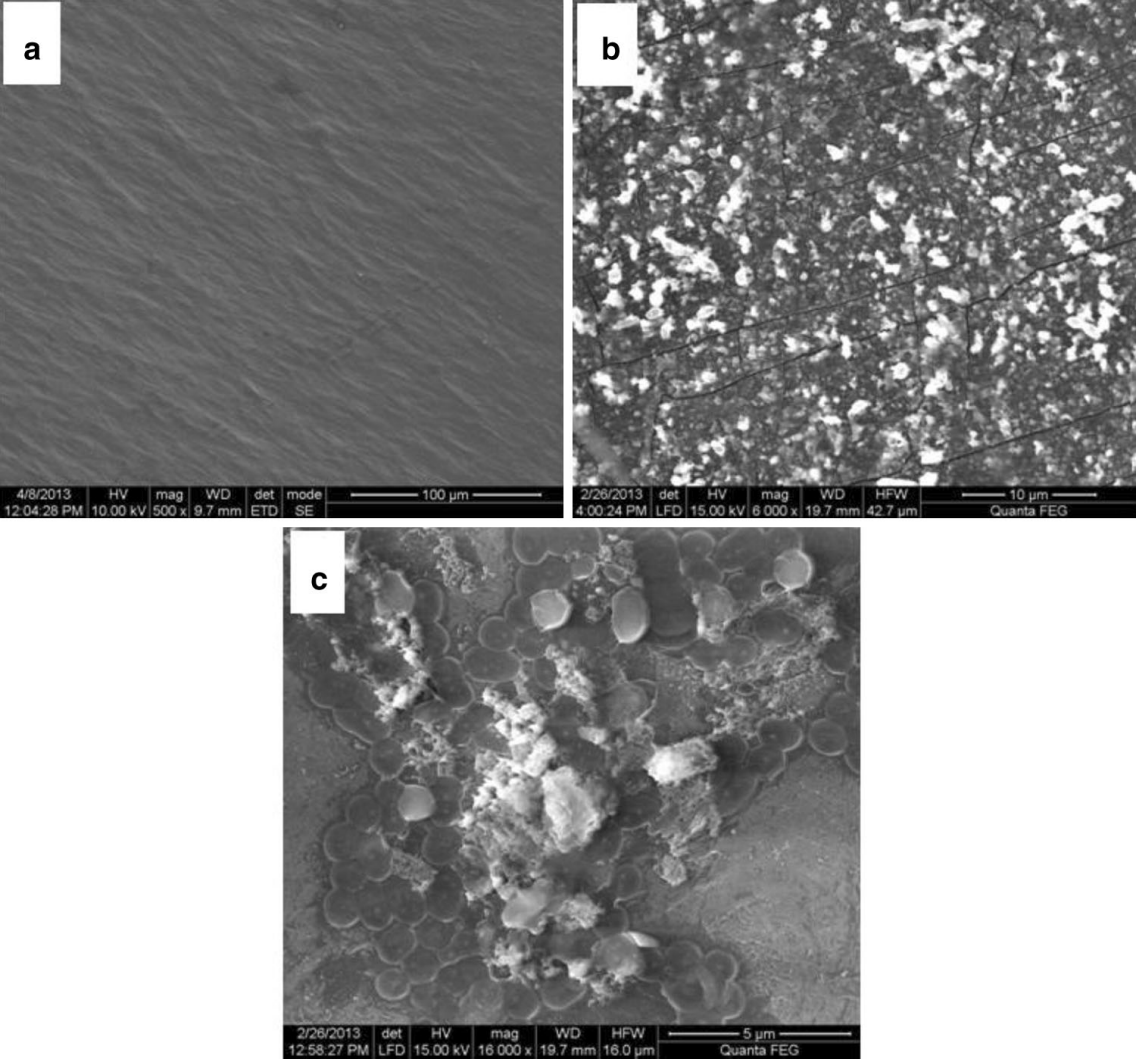
SEM micrograph of LDPE film before treatment as Control (**a**), LDPE film after treatment with BSM-1 (**b**), and LDPE film after treatment with BSM-2 (**c**)

While the pH change, weight reduction, mineralization level and absorption spectra provide solid evidence of polymer biodegradation, the changes of surface of LDPE films were elucidated by SEM. Control sample has an appearance of smooth surface having no pits, cracks or any particles attached on the surface (Fig. 3a). In the case of LDPE film treated with the bacterial isolate BSM-1, it was found that several cracks on the surface developed after 60 days of treatment. Simultaneously, microbes were also noticed on the film surface indicate its strong adhering capabilities as well as LDPE utilization capacities (Fig. 3b). The film treated with the bacterial isolate BSM-2 found to have bacterial attachment on higher rate as compared to BSM-1. Clear mark of degradation can be seen at places where initially microbes were attached along with the pockets and pits around (Fig. 3c). For both the strains, at different places on the surface several colonies forming biofilm can be observed.

#### FTIR analysis

**Fig. 4:**
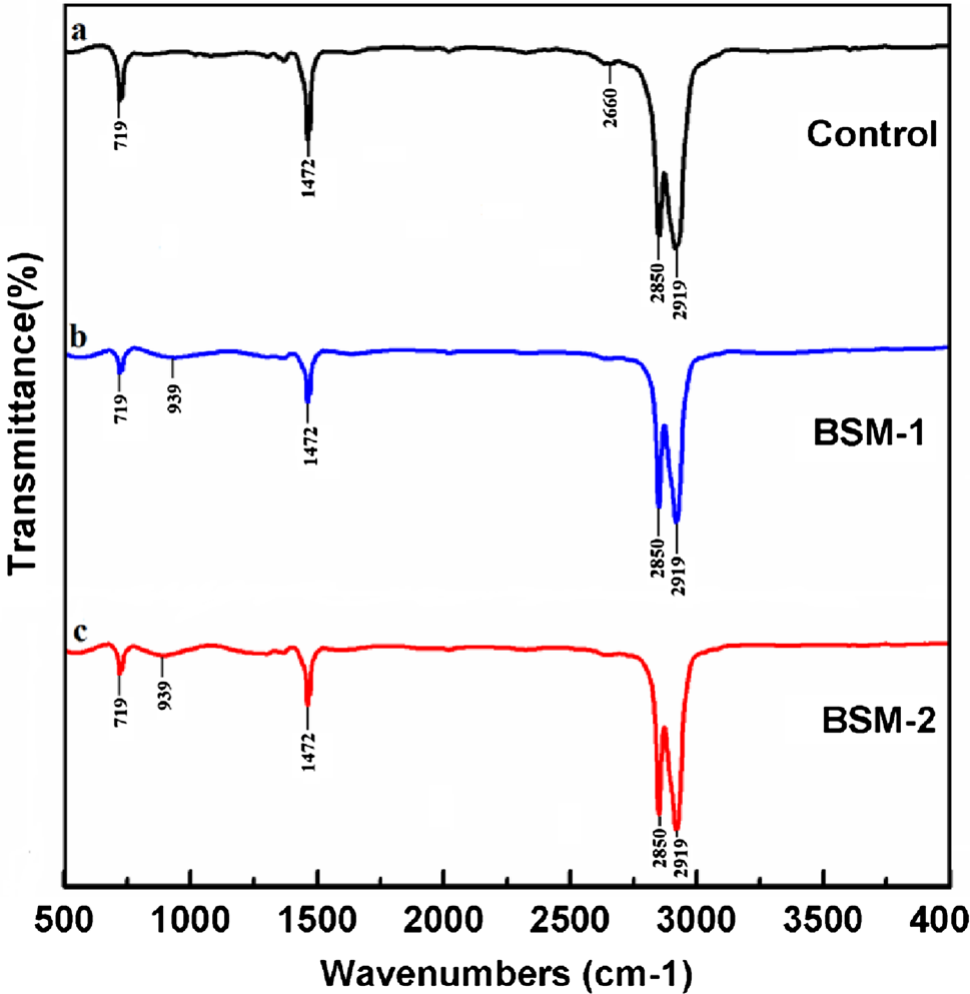
FTIR spectra of Control (*a*), treated with BSM-1 (*b*), and treated with BSM-2 (*c*)

FTIR spectroscopy is used as analytical technique in many biodegradation studies (Kiatkamjornwong et al. 1999; Klrbas et al. 1999; Arboleda et al. 2004; Drímal et al. 2007). It is a useful tool to determine the formation of new or disappearance of functional groups. So degradation products, chemical moieties incorporated into the polymer molecules such as branches, co-monomers, unsaturation and presence of additives such as antioxidants can be determined by this technique. Control spectra of polymer film (not treated with microbes) displayed a number of peaks reflecting the complex nature of the LDPE (Fig. 4a). There was a variation in the intensity of bands in different regions when test samples (after incubation with microbes, BSM-1 and BSM-2) were analyzed (Fig. 4b, c). For control spectrum, the characteristic absorption bands were assigned at 719 cm^−1^ (C–H bend-mono), 1,472 cm^−1^ (C=C stretch), 2,660 cm^−1^ (CHO stretch), and 2,919, 2,850 cm^−1^ (both due to C–H stretch). Significant and similar changes were found for both microbial strains. The peak at 2,660 cm^−1^ corresponds to CHO stretching vibration that has been disappeared in case of BSM-1 and 2 while new band has been observed at 939 cm^−1^ (O–H bend) which supports the depolymerization activity of the microbial isolates. The strong absorption peaks at 719 and 1,472 cm^−1^ became weaker after microbial treatment. In addition, the intensity of those peaks reduced more in case of BSM-2 than BSM-1 whereas peaks at 2,919 and 2,850 cm^−1^ became sharper in the treated sample than the control one, here also the same microbial activity pattern was seen. The change in the peak values of almost all functional groups supporting the conformational change on polymer surface.

## Conclusion

The problem of plastic pollution is now really a mess for mankind. There is no part of the world untouched from its impact. In the present era of globalization some stress must be given to plan safe disposal of products before making it commercial. Making science to the leap and forgetting the other side of coin lead to such conditions. In the present study, two isolated strains of *Bacillus amyloliquefaciens* were found to be useful for the biodegradation which is first time reported with applicable evidences. This biodegradation approach is safe and eco-friendly. The results showed a promising hope to degrade LDPE faster rate than to be degraded naturally.
